# Impacts of the US southeast wood pellet industry on local forest carbon stocks

**DOI:** 10.1038/s41598-022-23870-x

**Published:** 2022-11-14

**Authors:** Francisco X. Aguilar, Houston Sudekum, Ronald McGarvey, Benjamin Knapp, Grant Domke, Consuelo Brandeis

**Affiliations:** 1 Department of Forest Economics, Swedish University of Agricultural Sciences, 90736 Umeå, Sweden; 2 Tulane University, New Orleans, LA 70118 USA; 3 IESEG School of Management, 59000 Lille, France; 4 School of Natural Resources, University of Missouri, Columbia, MO 65211 USA; 5 Northern Research Station, United States Forest Service, United States Department of Agriculture, St. Paul, MN 55108 USA; 6 Southern Research Station, United States Forest Service, United States Department of Agriculture, Knoxville, TN 37919 USA

**Keywords:** Sustainability, Forestry, Energy policy

## Abstract

We assessed the net impacts of a wood-dependent pellet industry of global importance on contemporaneous local forest carbon component pools (live trees, standing-dead trees, soils) and total stocks. We conducted post-matched difference-in-differences analyses of forest inventory data between 2000 and 2019 to infer industrial concurrent and lagged effects in the US coastal southeast. Results point to contemporaneous carbon neutrality. We found net incremental effects on carbon pools within live trees, and no net effects on standing-dead tree nor soil pools. However, we found concurrent lower carbon levels in soils, mixed effects associated with increased procurement pressures and large mill pelletization capacity, and possible spillover effects on standing-dead tree carbon pools beyond commercial procurement distances. There is robust evidence that although some trade-offs between carbon pools exist, the wood pellet industry in this particular context and period has met the overall condition of forest carbon neutrality.

## Introduction

Nations around the world are adopting strategies to decarbonize their economies^1,2^. One decarbonization pathway is to substitute fossil fuels with biological resources in the generation of energy as illustrated by the European Union (EU) Bioeconomy Strategy and its Renewable Energy Directives^1,3,4^. Bioenergy—energy generated from biomass—is the EU28 (EU27 and Great Britain) largest renewable energy source with woody biomass procured from forests as the dominant biofuel in the generation of heat and power^5^. Worldwide, the EU28 is the biggest market for pelletized wood used as biofuel—internal trade of wood pellets more than tripled and imports into the EU28 grew seven-fold over the 2009–2019 period following adoption of the Renewable Energy Directives^3,4^. In 2020 the US was the world’s top producer (20%, weight) and exporter (25%, weight) of wood pellets, and the leading extra-regional supplier of wood pellets to the EU28^1^. US exports to the EU28 have grown 12-fold over the 2009–2019 period to reach 6.8 million Mg (1 Mg = 1 metric ton)^6^. Global wood pellet production topped 42 million Mg and their trade value surpassed US$4.3 billion in 2020^7^.

The capacity of biofuels to contribute to the decarbonization of the energy sector is inexorably linked to their procurement not depleting land carbon (C) stocks^8,9^. However, there are divergent views on this premise^10–13^ and robust empirical analyses testing it are lacking. Current understanding of wood-dependent biofuel industry effects on local forest C stocks has focused on market projections^12,13^ and state-of-knowledge syntheses^14,15^ with few empirical evaluations^16–18^. Empirical assessments are scarce partly due to the complexity of discerning the impacts of a wood-dependent biofuel industry that overlaps other economic sectors, social actors, and natural disturbances^15,18^.

**Figure 1:**
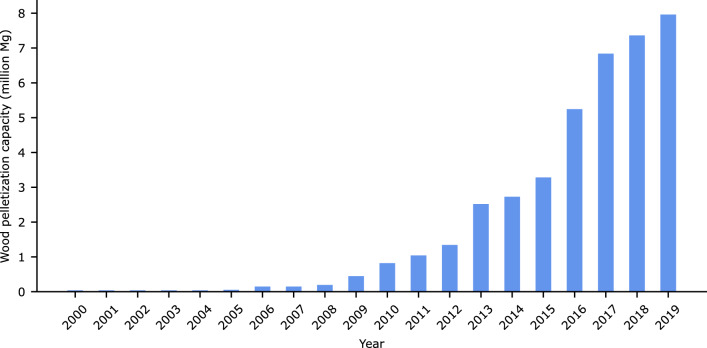
Total wood pelletization capacity in the US coastal southeast, 2000–2019. *Source*: Adapted from Forisk^19^.

Here, we used a post-matching difference-in-differences (DiD) approach to assess whether an industry that pelletizes woody biomass has affected total C stocks and individual component pools within live trees, standing-dead trees, and soils. We tracked C stocks in national forest inventory (NFI) plots located on private and public lands suitable for commercial management (timberland) sampled over the 2000–2019 period. During this period wood pellet annual manufacturing capacity grew from 40.823 thousand Mg to 6.652 million Mg in the US coastal southeast states of Alabama, Georgia, Florida, Mississippi, North Carolina, South Carolina, and Virginia (Fig. 1). We identified NFI plots located within prevalent commercial procurement distances measured by wood pellet mill-centered, tortuosity-adjusted geodesic radii, and across extended radii to examine possible spill-over impacts. We tested concurrent, lagged, and net contemporaneous industry effects—respectively denoting impacts within current year, delayed impacts at 5-year intervals, and net impacts of concurrent and lagged effects—on total C stocks and component pools. We expected to be able to statistically detect industry effects on local forest C stocks given the sharp rise in wood pellet manufacturing but were ambivalent as to their directional impacts.

Our contributions are three-fold. First, we analyzed industrial spatio-temporal effects on local timberland C stocks after controlling for the non-random siting of wood-using facilities. To our knowledge, this is the first post-matching DiD assessment of the impacts of a wood-dependent industry on local forest C stocks using NFI data. It expands on recent evaluations of the wood pellet industry’s effects using landscape-based estimates conducted in the US^16,17^, and remote-sensed analyses completed in Europe^18^. Second, our analytical framework offers an empirical alternative to evaluate systematic compliance with EU mandates to monitor forest C stocks in areas where woody biomass is procured for bioenergy^3,8^. We robustly examined our findings by using different algorithms to match NFI plots within and outside procurement radii, and confirmed general trends from a subsample of states where the industry had the largest expansion in capacity to-date. We also tested effects of mills’ large manufacturing capacity, and procurement pressures proxied by the number of procurement radii overlapping an NFI plot. Third, our results contribute to an enhanced understanding of C fluxes near wood-dependent industries. Of particular novelty, we estimate C in soils—an understudied pool—from empirical observations in our assessment of individual and total C stocks^20^.

## Methods

**Figure 2:**
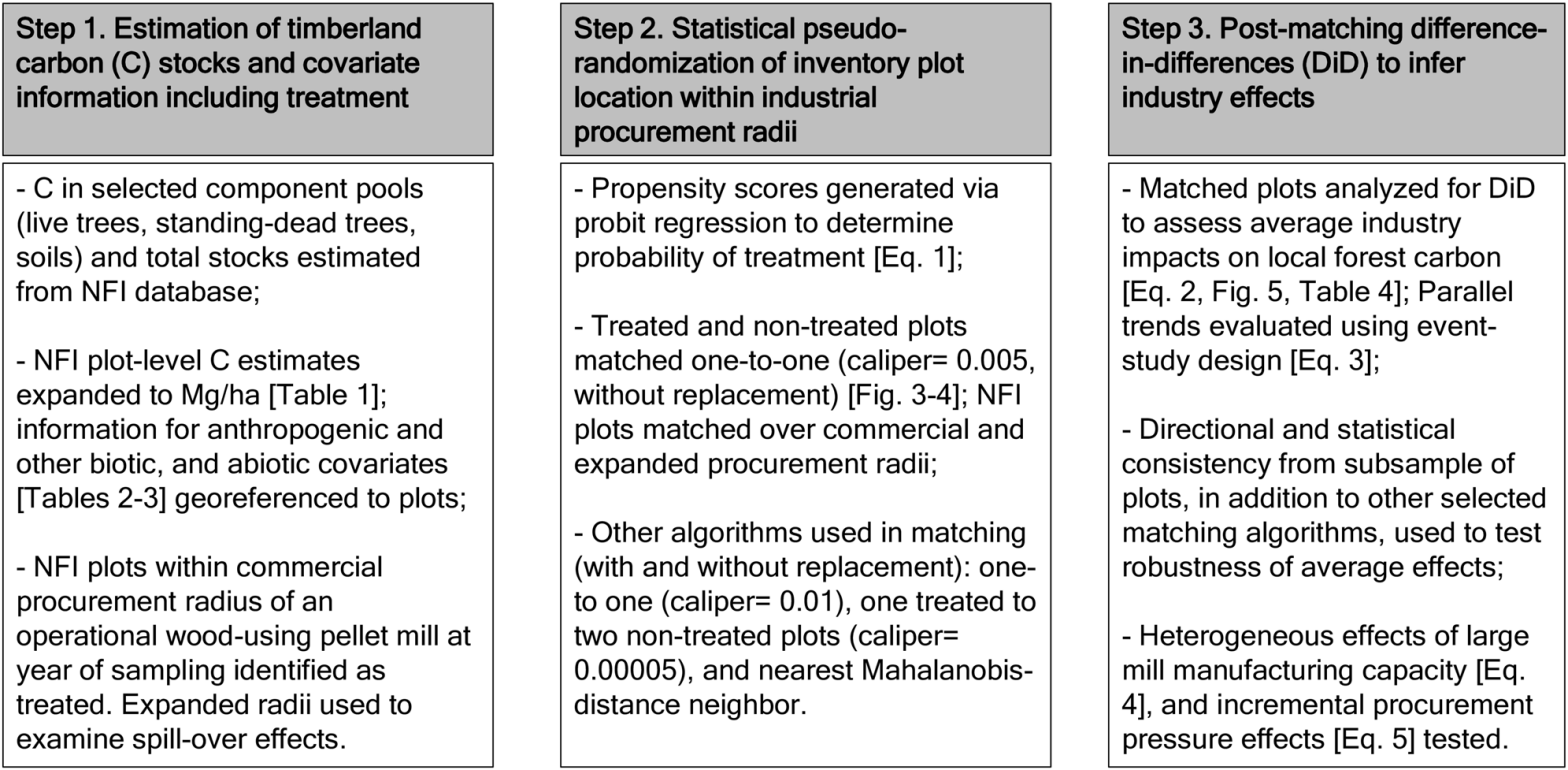
Methodological steps followed to estimate industrial impacts on local forest carbon component pools and total stocks.

Our empirical methods (Fig. 2) included three main steps: (1) estimation of timberland C stocks and covariate information across our study region; (2) statistical pseudo-randomization of NFI plots located within industrial procurement radii; and (3) post-matching estimation of average wood pellet mill industrial effects based on DiD panel regressions. Supporting analyses included examination of parallel trends prior to DiD, robustness checks for main effects, and evaluation of heterogeneous industry effects. Steps 1 and 2 were conducted in Python^21^, with final estimation completed in Stata Version 15^22^. Maps were generated using QGIS^23^.

### Estimation of timberland carbon stocks and covariate information including treatment

Estimates of C stocks above and belowground in live trees and standing-dead trees, and soils were obtained from plots sampled over the period 2000–2019 by the US Department of Agriculture Forest Service’s Forest Inventory and Analysis NFI program. Whether on public or privately owned lands, our sample included all NFI plots on timberlands (forestland capable of producing in excess of 1.4 m^3^ of industrial wood per ha per year and not legally withdrawn from timber production, with a minimum area classification of 0.41 ha)^24^ inventoried at least twice during our time period with no fewer than one observation recorded during or after 2005. Except for C in soils (available on request at this time—and eventually in the NFI database), all estimates are publicly available. Our compilation of soil C estimates^20^ relied on soil organic C observations from the NFI and auxiliary site and climate variables representing soil forming factors. These are used in US greenhouse gas reporting under the United Nations Framework Convention on Climate Change and better characterize localized soil conditions than current NFI information not meant for estimation at specific locations^20,24^. Extracted NFI plot-level information included data on tree-level measurements (e.g., number of live trees, standing-dead trees, biomass volume, stocking), as well as site characteristics (e.g., evidence of fire or weather damage) and assigned forest conditions (e.g., forest type). Details on NFI program sampling design, inventory procedures, and estimation of attributes are available online^24^. Descriptions of selected C stocks and total carbon (obtained by aggregating selected and other remaining C stock) are presented in Table 1. Per-hectare values were obtained using NFI expansion factors and aggregated based on plot sequence numbers, with estimates proportionally adjusted if less than 100% of a plot was classified as timberland^25^.

**Table 1:** Description of selected component pools and total carbon forest stocks within timberlands of the US coastal southeast.

Carbon pools	Description	Mean (mg/ha)	SD
Live-tree carbon	Carbon in the above- and below-ground portions of the tree, excluding foliage, of live trees ≥ 2.54 cm DBH/DRC	66.816	48.504
Standing-dead tree carbon	Carbon in the above- and below-ground portions of the tree, excluding foliage, of standing-dead trees ≥ 12.7 cm DBH/DRC	1.319	3.368
Carbon in soil organic material	Carbon in fine organic material below the soil surface to a depth of 1 m estimated from a model using soil forming factors^20^. Does not include roots. This attribute is a component of the US Environmental Protection Agency’s Greenhouse Gas Inventory and was obtained from a model developed directly from Phase 3 measurements of soil attributes in the US Department of Agriculture Forest Service’s Forest Inventory and Analysis NFI program	67.152	23.490
Total carbon	Carbon aggregated from forest plot measurements at tree-level (e.g. C in live and standing-dead trees) and at condition-level (C in down and dead trees, C in soil organic material, C in litter, and above- and below-ground carbon in the understory^‡^)	159.148	77.526

DBH: diameter at 137 cm above ground. DRC: diameter at root collar.

Mean and standard deviations derived from 39,882 national forest inventory plots in the original sample. Estimates were compiled at the inventory plot-level based on tree, condition and plot information. Plot-level timberland estimates were obtained from conditions contained within a plot. Non-timberland conditions were excluded, and plot level estimates were expanded proportionally. Live-tree and standing-dead tree carbon estimates were derived entirely from tree-level measurements on timberland conditions and expanded proportionally to the timberland portion of a plot. Other NFI-derived estimates were aggregated from condition to plot level.

^‡^Includes carbon in above- and below-ground portions of seedlings and woody shrubs as reported by NFI aggregated to carbon of organic material on the forest floor, including fine woody debris, humus, and fine roots in the organic forest floor layer above mineral soil.

Covariate information was distinguished between abiotic, biotic (Table 2), or of anthropogenic origin (Table 3)^15,26,27^. Assignment of several explanatory variables to an NFI plot (e.g., whether it sits within procurement radii of the wood products industry) required georeferencing, made possible by available plot-level latitude and longitude coordinates. It must be noted that plot locations in the NFI database are systematically ‘fuzzed’ or ‘swapped’ to protect the privacy of landowners and the integrity of the data collection process^25^. The ‘fuzzing’ process involves randomly relocating most plots within 0.8 km of their true location. The ‘swapping’ process occurs on 0–10% of forested plots, only for those that fall within private land^25^, and consists of exchanging coordinates with another similar plot within the same county. This random relocation process can have discernible effects in the estimation of forest conditions within small areas but any systematic bias on timberland attributes measured over a large geographic area, such as the procurement radii evaluated over the entire US southeast coastal region, are reportedly negligible^28^. Other attributes recorded at the county-level are very unlikely to carry any statistical noise from the fuzzing and swapping process^29^.

**Table 2:** Abiotic and biotic (non-anthropogenic) covariates used in the study of selected component pools and total carbon stocks in forest inventory plots located within timberlands of the US coastal southeast.

Covariate	Units	Spatial level	Mean	SD	Source
Evidence of fire damage since previous measurement	Yes = 1; Otherwise = 0	NFI plot	0.052	*n.a*.	^24^
Evidence of weather damage (other than fire), since previous measurement	Yes = 1; Otherwise = 0	NFI plot	0.020	*n.a*.	^24^
Evidence of insect or disease disturbance since previous measurement	Yes = 1; Otherwise = 0	NFI plot	0.022	*n.a*.	^24^
Palmer drought severity index	Index	NFI plot	− 0.488	2.106	^24^
Shannon’s diversity index (large-diameter): Meets diameter threshold for sawtimber-size trees; ≥ 22.9 cm (≥ 9 inches) for softwoods and ≥ 27.9 cm (≥ 11 inches) for hardwoods	Index	NFI plot	1.149	0.703	^24^
Shannon’s diversity index (small-diameter): Tree size classification at midpoint with diameter ≥ 12.7 cm (≥ 5 inches) and less than the sawtimber-size threshold	Index	NFI plot	0.803	0.618	^24^
Ecological forest type group*	Forest Inventory and Analysis Program’s group number: 1 = plot within a group, 0 = otherwise	NFI plot	Group 1 = 0.387 Group 4 = 0.121 Group 5 = 0.340 Group 6 = 0.107 Group 7 = 0.003 Group 8 = 0.300 Group 9 = 0.013	*n.a*.	^24^

*n.a.* Not applicable to categorical variables.

*Forest types adapted from^25^ where 1 = ‘White/Red/Jack Pine Group; Spruce/Fir Group, and other NFI 100-forest type codes’^24^; 4 = ‘Oak/Pine Group’; 5 = ‘Oak/Hickory Group’; 6 = ‘Oak/Gum/Cypress Group’; 7 = ‘Elm/Ash/Cottonwood Group’; 8 = ‘Maple/Beech/Birch Group’; 9 = ‘Alder/Maple Group’. No NFI plots belonging to groups 2–3 found in our sample area. Time variation possible due to differences in measurement between inventory cycles.

**Table 3:** Anthropogenic covariates used in the study of selected component pools and total carbon stocks in forest inventory plots located within timberlands of the US coastal southeast.

Covariate	Units	Spatial level	Mean	SD	Source
Location within procurement radii of the wood pellet industry, year of inventory (*t*)*	Yes = 1; Otherwise = 0	NFI plot	0.276	*n.a.*	^24^
Location within procurement radii of the wood pellet industry, previous inventory year (*t* − 1)*	Yes = 1; Otherwise = 0	NFI plot	0.123	*n.a.*	^24^
Location within procurement radii of the wood pellet industry, two previous inventory years (*t* − 2)*	Yes = 1; Otherwise = 0	NFI plot	0.036	*n.a*.	^24^
Geodesic distance to nearest operating pulp mill^†^	Km	NFI plot	58.247	34.793	^36^
Geodesic distance to nearest power plant generating at least 25,000 MWh from wood fuels^†^,*	Km	NFI plot	155.385	81.081	^37^
Geodesic distance to the nearest port trading forest products^†^,₸	Km	NFI plot	109.480	71.635	^38^
Road density₸	Km/ha	County	0.004	0.002	^39^
Population density	Number of county-level inhabitants per ha	County	8.200	13.063	^40^
Artificial forest regeneration	Evidence of artificial regeneration = 1; Otherwise = 0	NFI plot	0.270	*n.a*.	^24^
Land tenure (ownership) categories	Private ownership = 1; Otherwise = 0	NFI plot	0.911	*n.a*.	^24^
Location within US coastal southeast state₸	Alabama, Florida, Georgia, Mississippi, North Carolina, South Carolina, and Virginia: 1 = plot within a state, 0 = otherwise	NFI plot	AL = 0.196; FL = 0.074; FA = 0.225; MS = 0.091; NC = 0.157; SC = 0.138; VA = 0.120	*n.a*.	^24^

*Denoted concurrent and lagged ‘treatment effects’ identified by concentric circles of 48.28 km (30 imperial miles), and 80.47 km (50 imperial miles) of radii from a wood pellet mill’s centroid if its annual pellet manufacturing capacity in inventory year ‘*t*’ was, respectively, below or at least 100 000 Mg. ^†^Distances estimated from NFI plot centroids.

₸Time-invariant during our observed sampling period.

Treatment was identified by whether a NFI plot was within commercial industrial procurement distances of operational wood-using pellet mills at year of sampling. We drew concentric circles delimited by radii of 48.28 km (30 imperial miles), and 80.47 km (50 imperial miles) from a mill’s centroid if its annual pellet manufacturing capacity was, respectively, below or at least 100 thousand Mg to identify treated plots. The selected radii correspond with prevalent maximum travel distances and regional road tortuosity used in previous definitions of wood procurement areas^30–32^. Travel distances were validated with NFI’s Timber Products Output mill-level surveys and justified the longer procurement radius used for larger-capacity mills^33^. Defined procurement radii to assess industrial effects on forests have been applied to the wood product^16,17^ and other industries^34,35^. We explored the effects of adjusting procurement radii by extending them by 20 km as part of our assessment of spill-over effects.

### Statistical pseudo-randomization of forest inventory plot location within industrial procurement distances

Estimation of net industry effects on timberlands’ contemporaneous C levels first controlled for the non-random location of wood-using pellet mills. The siting of land-based resource-dependent facilities is a non-random process particularly when a large proportion of input expenditures arise from procurement and transportation costs of localized resources, and manufactured products have relatively low value-to-weight ratios^41^. The non-random siting process is well reported in wood pellet industry case studies^42^ and optimization applications^43^.

We relied on propensity score matching (PSM) to pseudo-randomize treatment and reduce initial observable heterogeneity in explanatory factors, leading to more accurate panel model estimations^44,45^. Use of PSM to pseudo-randomize plots has only recently been applied to NFI data^46,47^. PSM allowed us to identify inventory plots with conditions that could have made them as statistically-likely as those within procurement radii to be treated, yet were not. The estimation of the probability of an NFI plot in our sample (*n*) being within the procurement radius of a wood pellet mill was given by:

1 $${\text{Prob}}_{i} \left( {R = 0,1|M_{i} } \right),\quad i = 1,2 \ldots n;$$

where *R* takes on value of ‘1’ if the *i*th plot was located within the procurement radius of a wood pellet mill at any point during our sample period, and ‘0’ otherwise. Industry location theory and empirical evidence were our basis for choosing PSM covariates in vector *M*_*i*_^42,48,49^. These included: (a) Geodesic distance to nearest port trading forest products, (b) county’s road density, (c) land ownership (private or other), (d) geodesic distance to nearest wood-using power plant, (e) geodesic distance to nearest pulp mill, and (f) state- and (g) forest group type-specific effects. Respectively, the first three covariates proxied conditions that directly affect production costs including delivery to markets (hauling to trading ports), local transportation infrastructure (affecting ease of raw fiber procurement and delivery of manufactured pellets), and transaction costs (in this particular context, costs of procuring timber from privately held timberlands are on average lower than other ownerships partly due to contractual and administrative expenses). The next two covariates captured local competition by other industries procuring similar types of woody biomass (power plants burning wood, pulp mills). The last two covariates controlled for effects specific to a state and forest ecological subsection such as policies and regulations, and ecological conditions, respectively^50,51^. Vector *M*_i_ also included an intercept term. Geodesic distances were natural log-transformed to capture non-linear associations^52,53^. Incorporation of ecological and socio-economic variables has been empirically explored when using NFI plot information to test anthropogenic interventions on forest conditions^54^, and the inclusion of explicit spatial dimensions when matching NFI plots has enhanced PSM performance^46^. PSM variables were time-invariant with the exception of distances to nearest wood-using power plant and pulp mill, in which cases we used the average minimum distance over the sample period.

PSM scores for NFI plots within both commercial and extended procurement radii were estimated using a probit function with heteroskedasticity-robust standard errors^45^. We matched (with and without replacement) each plot of *R* = 1 with a non-treated plot using a caliper of 0.005. We used other matching algorithms including single matching with caliper 0.01, matching with two non-treated plots (caliper: 0.00005), and Mahalanobis-distance nearest neighbor. In the Results section we present findings following one-to-one (caliper: 0.005, without replacement) matching due to its performance in terms of reduced bias and conservation of original sample size after matching. Core PSM performance measures across matching algorithms are disclosed in Supplementary information (Tables S1–S2).

### Post-matching difference-in-differences to infer industry effects

Carbon in selected component pools (live-trees; standing-dead trees; soil organic material) and total stocks at the *i*th NFI plot of the *s*th forest type group in year *t* were modeled post PSM as:

2 $$C_{ist} = X_{it} \beta + D_{i,t - l} \delta + c_{i} + \omega_{s} + \gamma_{t} + \varepsilon_{ist} ,$$

where *X* is a matrix capturing time-variant covariates (excluding wood pellet industry effects); *D* is a matrix denoting whether a plot was within the wood pellet industry procurement radii at inventory year *t − l* (*l* = 0, 1, 2) for concurrent and lagged average effects; *c*, *ω* and *γ* capture plot-, forest type- and year-effects; and *ε* denotes a random error. Our model specification included NFI plot-level (*c*_*i*_) fixed effects after Breusch-Pagan Lagrange multiplier and Hausman test-statistics, respectively, favored *i*th-specific effects over pooled OLS estimation and their inclusion as fixed (over random) terms^55^. Standard errors were clustered at the plot level using the Delta method. Estimation of Eq. (2) and other models described in this section included an intercept term. Estimated regression parameters are found in vectors *β*, *δ*.

The direction and statistical significance of coefficients in *δ* measured average wood pellet industry effects. We assessed net industry effects using F Chow-tests of joint statistical significance of parameters in *δ*. Concurrent (*t*) and lagged (prior inventory year ‘*t − *1’, and two inventory years prior ‘*t* − 2’) captured treatment effects. We refer to their corresponding signals on contemporaneous C levels as lagging on 5 and 10 years (NFI plots were sampled most frequently every 5 years as per NFI program design and every 5.64 years on average). It is worth noting that our choice of using operational wood pellet mills (e.g., over actual production) to assess average treatment effects is rooted on rent principles, as an industry affects land net present value by the stream of expected future revenues, and not only on production in a particular year^53^. By extension, any form of anticipatory harvest behavior prior to the beginning of pelletization operations could have impacted land rents and confounded treatment effects^56^. However, this is very unlikely to be a source of bias in our NFI data. The period from announcement of intent to construct a wood pellet mill to actual operations commonly takes less than a year^57^ in our study region, making it unlikely to have had anticipatory effects reflected in NFI plot conditions re-sampled every five years.

Tests to validate our post-matching DiD regression included examining parallel trends prior to a NFI plot being within a wood pellet mill’s procurement radius. Empirically, we investigated pre-trends through an event study design. C levels in selected pools and total stocks post PSM were modeled as:

3 $$C_{ist} = \mathop \sum \limits_{y = - 15}^{y = 19} \tau_{yit} + c_{i} + \omega_{s} + \gamma_{t} + \varepsilon_{ist} ,$$

where *y* denotes the number of years prior to and after being in the radius of an operating wood pellet mill, with *y* = 0 reflecting when the *i*th NFI plot was first treated. *τ* is a vector of respective coefficients. Upper and lower limits denote years before/after treatment reflect maximum values in our dataset post PSM. Standard errors were clustered at the plot level.

The robustness of our findings, in addition to applying different PSM matching algorithms prior to DiD, was checked by estimating parameters from a subsample of NFI plots. We selected NFI plots within the states of Alabama, Georgia, and Virginia, where the industry has had some of the largest expansion and where most NFI observations during our study period were. We also tested for heterogeneous industry effects by: (a) distinguishing between mills of large manufacturing capacity (≥ 100 thousand Mg/year) and those of smaller size to assess shifts caused by manufacturing capacity, and (b) counting the number of procurement radii overlapping an NFI plot to examine incremental procurement pressures. To test the former, we estimated:

4 $$C_{ist} = X_{it} \beta + D_{i,t - l} \delta + S_{i,t - l} \nu + c_{i} + \omega_{s} + \gamma_{t} + \varepsilon_{ist} ,$$

where *S*_*i,t*−*l*_ captures wood pellet mill size information (1 = NFI plot within the radius of a wood pellet mill of manufacturing capacity ≥ 100 thousand Mg/year, 0 = otherwise) at inventory year *t − l*, and *ν* is a vector of estimated coefficients. We calculated Chow F-statistics to test joint significance of main-effects coefficients in *δ*, and heterogeneous size effect coefficients in *ν*. To test effects of overlapping procurement radii we estimated:

5 $$C_{ist} = \mathop \sum \limits_{r = 1}^{R} V_{ri,t - l} \lambda + c_{i} + \omega_{s} + \gamma_{t} + \varepsilon_{ist} ,$$

where *V*_*ri,t*−*l*_ denotes the *r* number of overlaps per *i*th NFI plot from 1 to ‘*R*’ at inventory year *t*-*l*, and *λ* is a vector of estimated coefficients. We identified as many as five (*R* = 5) industry radii overlaps at inventory year *t* and *t* − 1, and as many as two at *t* − 2. Over extended radii, we identified as many as six overlaps at each *t* and *t* − 1, and as many as two at *t* − 2. The baseline category was our control of a NFI plot not being within the procurement radii of the wood pellet industry. We calculated Chow F-statistics to test joint significance of coefficients capturing single radius (*r* = 1), heterogeneous effects of industry radii overlap (*r* > 1) within vector *λ*, and net total industry effects (*r* ≥ 1).

## Results

### Timberland carbon stocks after propensity score matching

Results of the probit regression [Supplementary information, Table S3] showed that the probability of a plot being within commercial procurement radius of a wood pellet mill decreased with the longer distance from the nearest port (ρ coeff. = −0.335, *p* < 0.001). A similar, but only marginally significant association (ρ coeff. = −0.024, *p* = 0.105) was found with distance to nearest pulp mill. This weaker spatial correlation might be explained by the co-location of supply chains. Contrary, there was a direct association with distance to nearest wood-using power plant (ρ coeff. = 0.159, *p* < 0.001) possibly explained by how these two industries directly compete for similar low-cost wood fibers. A negative coefficient was found for the density (km/ha) of primary and secondary roads in the county of the *i*th plot (ρ coeff = −30.249, *p*  < 0.001), which might point to how above average region-wide road density also increases land opportunity costs as it is the case of more urbanized areas. We found a direct association with private ownership (ρ coeff. = 0.282, *p* < 0.001) over other types of ownership. Table S3 also shows respective results when the expected probability was that of a plot being located within extended procurement radii.

**Figure 3:**
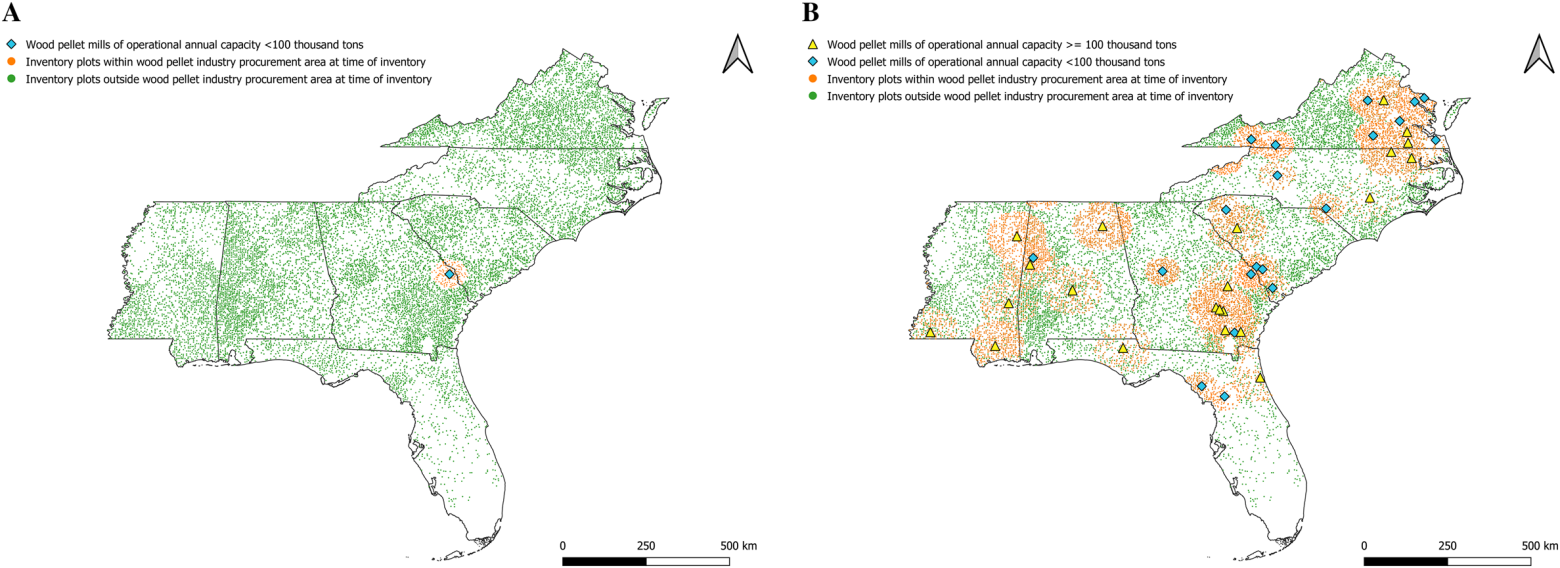
Location of national forest inventory plots on timberlands of the US coastal southeast. Including resampled plots between 2000 and 2019 and of operating wood pellet mills as of year (**A**) 2000 and (**B**) 2019, after propensity score matching (caliper 0.005, without replacement). Maps generated using QGIS Desktop 3.18.2, available online at https://download.qgis.org/downloads/.

**Figure 4:**
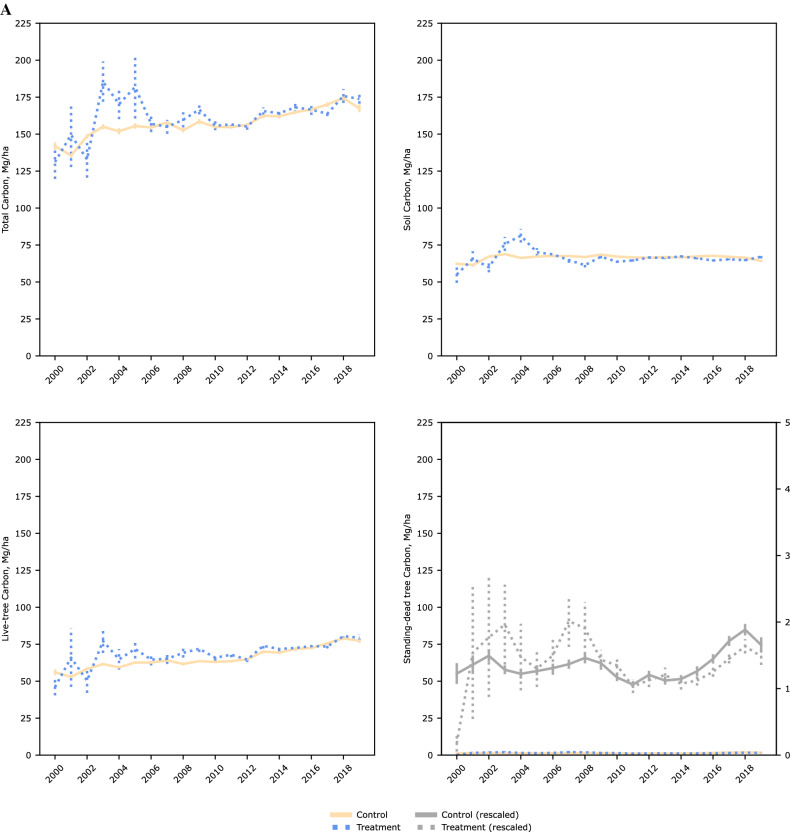

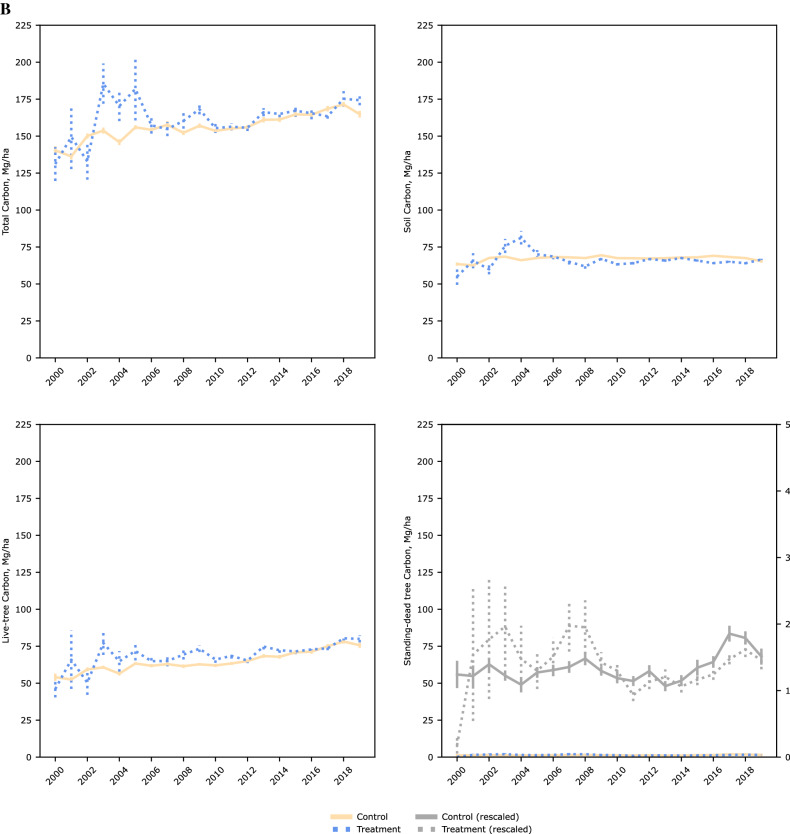

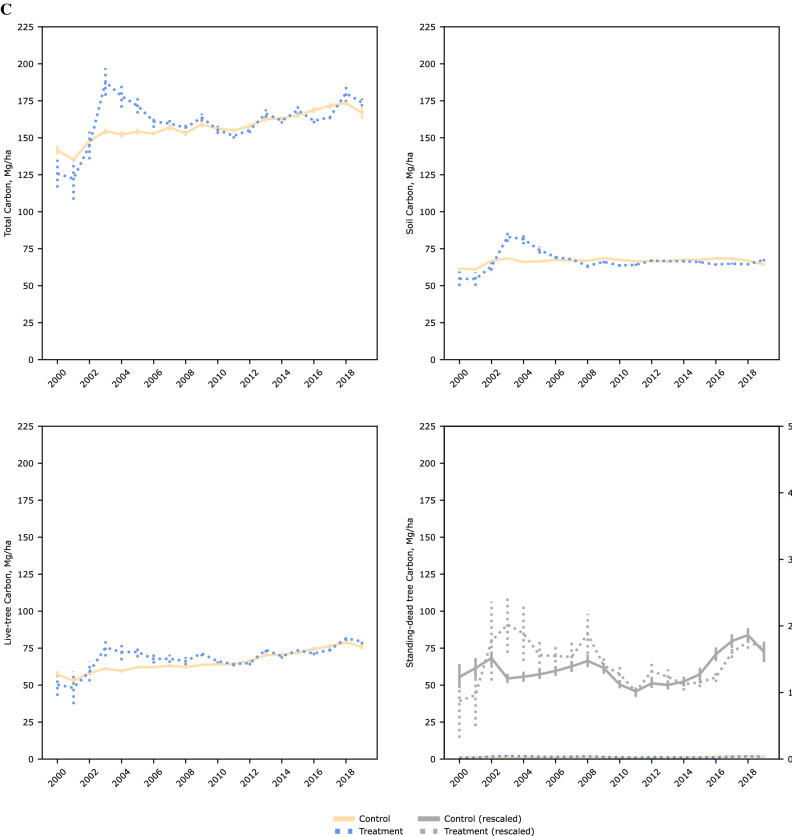
Annual mean estimates of selected component pools and total carbon stocks over inventory years (2000–2019). By (**A**) overall sample, and by treatment category after propensity score matching (caliper = 0.005, without replacement) within (**B**) commercial, and (**C**) extended procurement radii.

Figure 3 shows the NFI plots included in our analyses post PSM. Matching reduced bias across covariates particularly regarding distance to other wood-using industries (pulp mills and power-generating facilities using wood as a feedstock), and distance to ports trading forest products. Over our time period across timberlands of the US coastal southeast, there was a steady increase in total C levels as well as in live trees and standing-dead trees (Fig. 4). C within soils showed the least variability over time as expected. Tests of parallel trends [Supplementary information, Figure S1] prior to a NFI plot being within a wood pellet mill’s procurement radius using an event study design showed no systematic differences.

### Wood pellet industry impacts on local forest carbon stocks

**Figure 5:**
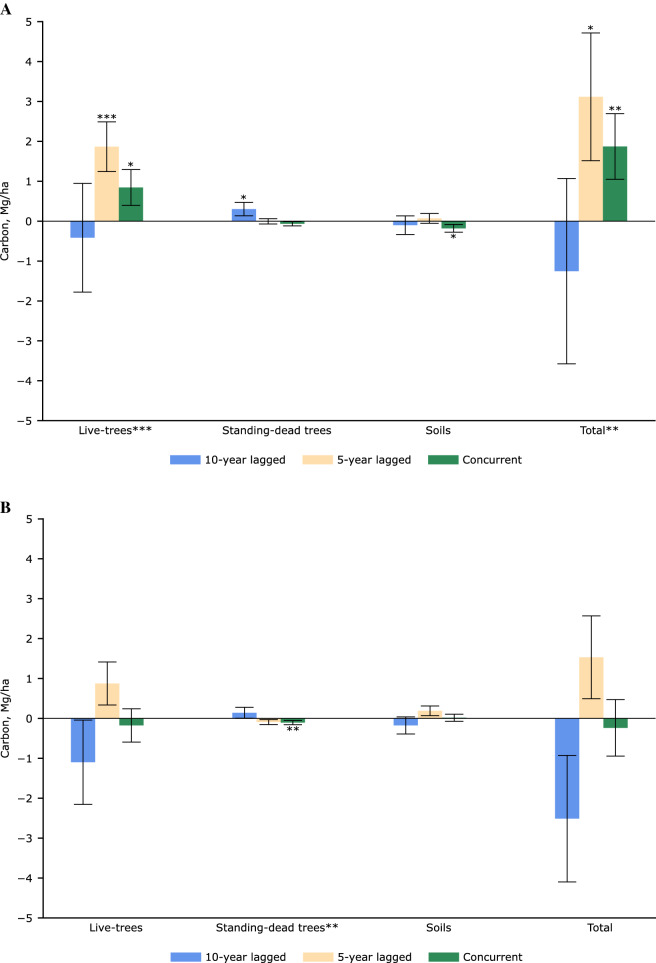
Estimated average concurrent and lagged effects of the wood pellet industry on selected pools and total C stocks in timberlands of the US coastal southeast. Results by (**A**) commercial [NFI plots = 14,342; Observations = 39,882], and (**B**) extended [NFI plots = 19,438; Observations = 52,895] procurement radii. Average effects inferred after propensity score matching (caliper = 0.005, without replacement) and fixed-effects panel regression. Bars denote robust inventory plot-clustered standard errors. Type-I errors (* < 0.10; ** < 0.05; *** < 0.01) of concurrent, lagged, and net effects on respective carbon stocks.

There was statistical evidence from a sample of 14,342 NFI plots that the wood pellet industry in the US coastal southeast affected contemporaneous levels of C in live trees (F-test: *p* = 0.009) and total forest C stocks (F-test: *p* = 0.035) within commercial procurement radii (Fig. 5A). We found no statistically discernible net contemporaneous effects within standing-dead trees (F-test: *p* = 0.172), nor soils (F-test: *p* = 0.214). Net industry effects identified across extended radii (Fig. 5B) estimated from a sample of 19,438 NFI plots suggests no statistical significance on C within live-trees (F-test: *p* = 0.242), soils (F-test: *p* = 0.387), nor total C stocks (F-test: *p* = 0.196), but an effect on standing-dead tree C pools (F-test: *p* = 0.044).

Within commercial procurement radii, total C stocks showed higher concurrent and 5-year lagged effects at an average of 1.871 Mg/ha (*p* = 0.023) and 3.116 Mg/ha (*p* = 0.052). There were average higher concurrent and 5-year lagged effects of 0.844 Mg/ha (*p* < 0.060) and 1.866 Mg/ha (*p* = 0.003), respectively, on C in live-tree pools; and 10-year lagged effects (0.302 Mg/ha, *p* = 0.070) on C in standing-dead trees. C in soils showed lower concurrent levels (− 0.180 Mg/ha; *p* = 0.061). Average industry effects identified over extended procurement radii point to no statistically significant concurrent nor lagged impacts on live-tree, soil C pools, or total stocks. However, there was a concurrent inverse effect (− 0.105 Mg/ha; *p* = 0.044) on the standing-dead tree C component pool.

Average industry effects relative to total and component C pools were modest (Table 4). Their absolute effects declined across extended procurement radii for total C stocks and live tree C pools, with the exception of 10-year lagged effects (although these were not statistically significant). Relative average effects on standing-dead trees were the largest amongst individual C component pools: their greatest statistically-significant effect (*p* < 0.10) show a 22.811% 10-year lagged increase within commercial procurement radii and a 7.530% concurrent decrease over extended radii. Soil C pools showed the lowest relative effects. Statistically significant (*p* < 0.10) mean relative effects on total stocks resulted in 1.135% concurrent and 1.890% 5-year lagged greater than average C within commercial procurement distances.

**Table 4:** Estimated average wood pellet industry effects relative (%) to mean component pools and total forest C stocks in timberlands of the US coastal southeast, by prevalent commercial and extended radii.

Procurement radii₸	Live trees		Standing-dead trees		Soils		Total	
	Commercial**	Extended	Commercial	Extended*	Commercial	Extended	Commercial*	Extended
(C, Mg/ha)^†^	(72.323)	(71.853)	(1.326)	(1.391)	(65.653)	(65.755)	164.858	164.104
Concurrent	1.167%*	− 0.245%	− 4.915%	− 7.530%**	− 0.275%*	0.027%	1.135%**	− 0.144%
5-year lagged	2.580%***	1.218%	− 0.367%	− 6.614%	0.105%	0.289%	1.890%*	0.933%
10-year lagged	− 0.574%	− 1.528%	22.811%*	10.188%	− 0.153%	− 0.268%	− 0.761%	− 1.530%

₸Joint significance of concurrent and lagged industry effects estimated from Chow F-statistics. ^†^Average of forest inventory plots located within respective procurement radii. Type-I errors: * < 0.10; ** < 0.05; *** < 0.01.

Parameters estimated after different matching algorithms and from a reduced sample (Supplementary information, Figures S2-S4) showed overall consistency of net contemporaneous neutrality in total C stocks; the largest effects were found in live tree component pools within commercial radii. Notably, across extended radii there was some apparent indication of spill-over effects in individual C pools, but no evidence of change in total C stocks. Tests of heterogeneous size effects (Supplementary information, Tables S4–S5) suggest there was no net effect on total C stocks either. But we identified a net effect on standing-dead tree pools (F-test: *p* = 0.071) within the commercial procurement radii of wood pellet mills of at least 100 thousand Mg of annual capacity. Although not jointly significant, we found lower 10-year lagged levels on live C pools (− 8.100 Mg/ha, *p* = 0.05). When we tested heterogeneous size effects over longer radii, we detected a net increase in soil C (F-test: *p* = 0.038). Heterogeneous effects due to the overlap of commercial procurement radii (i.e., a plot overlapped by more than one wood pellet mill’s procurement radius) suggest similar trends (Supplementary information, Tables S6–S7). Greater overlaps in commercial procurement radii were associated with a significant mean increase in C within live trees (F-test, *p* < 0.001), soils (F-test: *p* < 0.001) and total stocks (F-test, *p* < 001). It is worth noting that we also detected some negative impacts (e.g., less C in live trees − 7.225 Mg/ha, *p* = 0.05 with 5-year lagged effect of a 5th radii overlap). Overlaps of mills’ extended procurement radii was associated with less C in standing-dead trees (F-test: *p* = 0.020).

## Discussion

Industry effects on contemporaneous C levels in live trees are likely explained by timber-oriented management. This is the one C pool that is actively valued financially, creating an expected stream of net revenues that increase land rents within industrial procurement areas^58^. Higher land rents motivate the implementation of silvicultural practices to grow timber, thus yielding more C in live trees^14^. Our results corroborate market projections of how new bioenergy demand can coexist with continued net biomass growth within commercially procured areas^12,13,57^. Differences in units of observation and statistical models prevent direct comparisons, but our mean estimates fall within the average increase of 2 Mg/ha previously detected for large-scale wood pellet mills’ procurement landscapes operating for at least 10-years in the eastern US in the 2005–2017 period^16^. Noticeably, we found no statistically significant effects on live tree C pools across extended procurement radii (although other matching techniques showed marginally significant effects). This might be explained by localized sourcing of woody biomass within ‘wood baskets’ due to transportation comprising a large proportion of procurement costs^59^.

A land rent rationale might not be extended to C component pools that do not garner financial returns, but impacts could still be linked to timber-oriented management practices. In the case of standing-dead trees, this is consistent with past studies^16,17^ reporting no statistically discernible changes in this C pool across industrial procurement landscapes. A plausible explanation of 10-year lagged effects on C in the standing-dead trees component pool within commercial procurement radii might be the adoption of practices that retain a minimum number of standing-dead trees, tree crowns, and other woody debris during harvest. Every state in our sample has adopted such recommendations to address concerns over ecological impacts linked to additional woody biomass extraction exceeding prevalent harvesting and natural disturbances^60^. However, associated industry effects over extended procurement distances suggest a concurrent decline in C in the same standing-dead tree component pool. This might be indicative of an expansive industrial procurement footprint reportedly associated with fewer standing-dead trees^16,17^ yielding a decline in this C pool when compounded over a larger area.

In the case of C in soils, there is growing evidence that timber harvesting may lower these stocks^61–63^ although post-harvest practices such as reforestation can help restore them^64^. These might explain the concurrent statistically-significant lower levels of soil C within commercial procurement radii and 5-year lagged higher levels detected across extended radii. Anthropogenic perturbations can lead to changes in soil temperature and moisture and, in turn, influence microbial accessibility and activity by reducing C inputs from litter material and roots^64^. Differences in soil C levels can also be attributed to time-invariant natural factors such as forest type and parent material^64,65^ which we controled for in our estimation. Another analysis using a different set of C estimates from ours also found an inverse trend for the soil C component pool across similarly-defined commercial procurement areas^16^. Within these interpretations, it is important to stress that all soil C estimates carry inherent methodological uncertainty, that can challenge the attribution of mechanisms of C change^20,64,66^.

Two of the key features of our methodological approach were the statistical pseudo-randomization of NFI plots located within industrial procurement radii, and controlling for important covariates (anthropogenic, other biotic, and abiotic factors). Others have attempted different approaches to control for non-random wood pellet mill location including the selection of a counterfactual region^16,35^. We formalized this step when conducting a PSM before DiD analyses. It is also imperative to control for non-industrial factors that can affect forest C stocks in order to correctly tease out industry-related impacts. Here, we point to the statistical associations we found between fire and extreme weather damage (Supplementary information, Figure S5), both likely to intensify with a changing climate^67^. For instance, evidence of fire damage and extreme weather other than fire were associated with an average drop in C in live trees of − 1.675 Mg/ha (*p* = 0.033) and − 7.473 Mg/ha (*p* < 0.001), respectively. We also found a direct association between insect or disease disturbance and C in live (3.050 Mg/ha; *p* = 0.005) and standing-dead (2.335 Mg/ha; *p* < 0.001) trees. These results might be respectively indicative of how such disturbance is more likely to be detected with more abundant live biomass and associated with an increase in tree mortality. It is important to note that the estimates associated with these abiotic and biotic factors surpass the industrial effects detected in our research.

Our methodological approach using NFI data can be applied to assess the localized land C neutrality of any industry dependent on woody biomass. But we stress that the ultimate neutrality of an entire bioenergy system—not just the land sector—is contingent on many factors besides wood procurement. Previous studies have reported that wood-based energy systems could result in a wide range of effects on net C emissions^68–70^. For instance, life-cycle assessments of C emissions from land until power conversion show that electricity generated from woody biomass could yield as much as 83% reductions in net C emissions, or as high as 73% net increases, over coal usage. Net increases in emissions were detected when high energy intensive biomass supply-chains were modelled (e.g., drying biomass in kilns using fossil fuels)^68^. Others, after taking into account trans-Atlantic shipment of wood pellets manufactured in the US coastal southeast and combusted in the EU28, have still suggested net lower C emissions over fossil fuel based-systems. This is because the market value created by the pellet industry can keep C stocks growing by preventing land use change away from forests^12,59^.

On the whole, we found no evidence of a net decline in total contemporaneous local forest C stocks caused by the wood pellet industry in the US coastal southeast. This result suggests that wood pelletization in this particular context may contribute to decoupling bioenergy objectives and that no additional C emissions should be attributed to the land sector for national-level greenhouse gas accounting^8^. Our findings also point to discernible trade-offs, particularly net gains in C within live trees but lower C in soils within commercial procurement areas, likely due to more intensive timber management. While our assessment of net neutrality holds, continued evaluation of total C stocks and individual pools seems needed for at least two reasons. First, our results may be statistically robust but the 20-year period covered in our study is relatively short to measure sustainability trends in forestry. Second, wood pellets to-date remain a relatively small component of the array of wood products manufactured in the US coastal southeast (Supplementary materials, Figure S6) but it is one of the fastest growing sectors of the wood products industry. Future changes in harvesting pressures caused by expected increases in wood product manufacturing demand, in combination with other factors, could plausibly alter C dynamics and net stocks.

Regarding future research needs, empirical assessments could be expanded to study impacts beyond C and to other contexts. For instance, it will be important to better understand any causal effects on the complex socio-demographic landscape where the US coastal southeast wood pellet industry has emerged^71^. Assessments on how a growing wood pellet industry might affect local biodiversity or other land management objectives are merited. Within complex forested landscapes, changes in species composition across timberland could be supportive or detrimental to efforts aimed at enhancing the capacity of forests to cope with a changing climate^72^. It would also be valuable to assess whether forest C stocks are impacted by the wood pellet industry in other geo-political contexts. For example, wood pelletization in Viet Nam has quadrupled in production over a five-year span to become the world’s third largest exporter. Its wood pellets exports exceeded 3 million Mg in 2019^6^. Systematic assessments of forest C neutrality in the EU27, where the largest wood pelletization capacity worldwide is currently found, seem warranted to overcome concerns over potentially spurious associations with wood-dependent bioenergy industries^73^.

## Conclusions

We assessed impacts of the wood pellet industry on local forest C stocks within timberlands of the US coastal southeast, distinguishing between component pools in live and standing-dead trees, soils, and total stocks. Our estimates offer robust evidence that the wood pellet industry has met the overall condition of forest carbon neutrality. Hence, this industry could have contributed to decoupling objectives and no additional C emissions should have been attributed to the land sector in greenhouse gas accounting over the 2000–2019 period.

Our findings also point to discernible trade-offs (e.g., gains in C within live trees, lower C in soils within commercial procurement areas) with timber management as the most plausible mechanism behind such changes, and possible spill-over effects particularly amongst non-financially traded C pools (e.g. lower C in standing-dead trees). When testing heterogeneous effects, there was also some indication of mixed effects on C pools when distinguishing wood pellet mill size and procurement pressure intensified. Nevertheless, our empirical evidence suggests C neutrality in the US coastal southeast. The relative recent emergence of the wood pellet industry limits our capacity to point to long-term sustainability trends, and emphasize that findings are applicable to the procuring of wood for pelletization in our particular study context.

## Supplementary Information

Supplementary Information.

## Data Availability

The source code for our statistical analyses is available at https://dataverse.harvard.edu/dataverse/woodpelletindustry. The online repository includes sample datasets and code data to reconstruct datasets.
