# Supplementary material for: Impacts of the US southeast wood pellet industry on local forest carbon stocks
Source: Sci Rep. 2022 Nov 14;12:19449. doi: 10.1038/s41598-022-23870-x (PMC9663713; doi:10.1038/s41598-022-23870-x)
Supplement: Supplementary file 1 — Supplementary Information. [file 41598_2022_23870_MOESM1_ESM.pdf]

Supplementary information to:

IMPACTS OF THE US SOUTHEAST WOOD PELLET INDUSTRY ON LOCAL FOREST CARBON STOCKS

Table S1. Standardized bias for covariates using selected matching algorithms.

Table S2. Standard errors for covariates after matching using selected algorithms.

Table S3. Results of probit regression used to generate propensity scores.

Table S4. Wood pellet industry heterogeneous effects (manufacturing capacity) on selected C stocks within timberlands of the US coastal southeast, commercial procurement radii.

Table S5. Wood pellet industry heterogeneous effects (manufacturing capacity) on selected C stocks within timberlands of the US coastal southeast, extended procurement radii.

Table S6. Wood pellet industry heterogeneous effects (radii overlap) on selected C stocks within timberlands of the US coastal southeast, commercial procurement radii.

Table S7. Wood pellet industry heterogeneous effects (radii overlap) on selected C stocks within timberlands of the US coastal southeast, extended procurement radii.

Fig. S1. Estimated coefficients from event study design. Results to test parallel trends in treatment and control areas prior to treatment by (A) commercial and (B) extended procurement radii.

Fig. S2. Estimated average concurrent and lagged effects of the wood pellet industry on selected C stocks in timberlands of the US coastal southeast using different matching algorithms (commercial procurement radii).

Fig. S3. Estimated average concurrent and lagged effects of the wood pellet industry on selected C stocks in timberlands of the US coastal southeast using different matching algorithms (extended procurement radii).

Fig. S4. Estimated average concurrent and lagged effects of the wood pellet industry on selected C stocks within timberlands of the states of Alabama, Georgia, and Virginia.

Fig. S5. Estimated associations between selected abiotic and biotic factors and C stocks in timberlands of the US coastal southeast within commercial procurement radii.

Fig. S6. Roundwood production utilization, by product type in the US coastal southeast, 2011-2020.

Table S1. Standardized bias for covariates using selected matching algorithms.

		One-to-one caliper 0.005		Nearest neighbor		One-to-one caliper 0.01		One-to-two caliper 0.001	
Commercial radii (# plots)	Pre-matching (21644)	No-resampling (14342)	Resampling (12396)	No-resampling (14970)	Resampling (12397)	No-resampling (14352)	Resampling (12397)	No-resampling (15866)	Resampling (15866)
Geodesic distance to nearest port (ln)	23.331	12.588	12.777	11.950	12.777	12.578	12.777	18.234	18.234
Density of primary and secondary roads in the plot's county (Km/ha)	7.200	2.787	3.544	2.141	3.544	2.783	3.544	4.976	4.976
Private ownership (Yes =1; Otherwise = 0)	2.749	0.417	0.259	0.428	0.259	0.426	0.259	0.612	0.612
Geodesic distance to nearest wood-using biopower facility (ln)	6.892	3.470	5.079	2.754	5.079	3.441	5.079	5.390	5.390
Distance to nearest wood-using pulp mill (ln)	11.165	6.333	5.730	6.227	5.730	6.327	5.730	9.446	9.446
Extended radii (# plots)	(21644)	(19438)	(16423)	(21510)	(16424)	(19489)	(16426)	(19095)	(19095)
Geodesic distance to nearest port (ln)	13.757	8.433	9.148	13.276	9.151	8.459	9.151	11.805	11.805
Density of primary and secondary roads in the plot's county (Km/ha)	3.868	3.108	3.210	3.874	3.218	3.136	3.218	3.571	3.571
Private ownership (Yes =1; Otherwise = 0)	1.906	0.493	0.957	1.870	0.948	0.514	0.948	1.609	1.609
Geodesic distance to nearest wood-using biopower facility (ln)	10.241	9.003	9.227	10.251	9.219	8.967	9.219	9.976	9.976
Distance to nearest wood-using pulp mill (ln)	10.720	9.553	9.897	10.410	9.894	9.563	9.894	9.965	9.965

Table S2. Standard errors for covariates after matching using selected algorithms.

	Pre-matching		One-to-one caliper 0.005		One-to-one caliper 0.01		Nearest neighbor		One-to-two caliper 0.001	
Commercial radii	Treated	Control	Treated	Control	Treated	Control	Treated	Control	Treated	Control
Geodesic distance to nearest port (ln)	0.637	0.332	0.637	0.430	0.637	0.430	0.637	0.417	0.637	0.359
Length of primary and secondary roads in the plot's county (Km/ha)	<0.001	<0.001	<0.001	<0.001	<0.001	<0.001	<0.001	<0.001	<0.001	<0.001
Private ownership (Yes =1; Otherwise = 0)	0.003	0.001	0.003	0.002	0.003	0.002	0.003	0.002	0.003	0.001
Geodesic distance to nearest wood-using biopower facility (ln)	0.771	0.371	0.772	0.477	0.772	0.477	0.772	0.464	0.772	0.400
Distance to nearest wood-using pulp mill (ln)	0.242	0.181	0.242	0.222	0.242	0.222	0.242	0.216	0.242	0.193
Extended radii	0.538	0.354	0.636	0.365	0.634	0.365	0.540	0.354	0.557	0.356
Geodesic distance to nearest port (ln)	<0.001	<0.001	<0.001	<0.001	<0.001	<0.001	<0.001	<0.001	<0.001	<0.001
Density of primary and secondary roads in the plot's county (Km/ha)	0.002	0.002	0.003	0.002	0.003	0.002	0.002	0.002	0.002	0.002
Private ownership (Yes =1; Otherwise = 0)	0.616	0.396	0.671	0.405	0.669	0.405	0.617	0.395	0.624	0.396
Geodesic distance to nearest wood-using biopower facility (ln)	0.201	0.200	0.230	0.208	0.229	0.208	0.201	0.200	0.206	0.201

Table S3. Results of probit regression used to generate propensity scores (*n*= 21,644).

	Commercial procurement radii				Extended procurement radii			
**Variables†**	**Coef.**	**Standard error ‡**	**z-statistic**	***p*>|z|**	**Coef.**	**Standard error ‡**	**z-statistic**	***p*>|z|**
Intercept	0.015	0.090	0.190	0.860	-0.078	0.086	-0.908	0.364
Geodesic distance to nearest port (in natural logs)	-0.335	0.013	-25.076	<0.001	-0.267	0.014	-19.767	<0.001
Geodesic distance to nearest wood-using biopower facility (in natural logs)	0.159	0.015	10.500	<0.001	0.239	0.015	16.008	<0.001
Geodesic distance to nearest wood-using pulp mill (in natural logs)	-0.024	0.015	-1.619	0.105	-0.051	0.015	-3.463	0.001
Density of primary and secondary roads in the plot's county (Km/ha)	-30.249	5.352	-5.652	<0.001	-5.630	5.252	-1.072	0.284
Private ownership of the largest condition in the plot (Yes =1; Otherwise = 0)	0.282	0.030	9.427	<0.001	0.225	0.028	8.030	<0.001
	Degrees of freedom=18; Residual degrees of freedom= 21625; Log-likelihood: -13222 (*p*-value: <0.001)				Degrees of freedom=18; Residual degrees of freedom= 21625; Log-likelihood: -14413: (*p*-value: <0.001)			

†Categorical variables included for each state and forest group types during estimation.

‡Heteroskedasticity-robust standard errors.

Table S4. Wood pellet industry heterogeneous effects (manufacturing capacity) on selected C stocks within timberlands of the US coastal southeast, commercial procurement radii†.

	Live trees		Standing dead-trees		Soils		Total	
**Main effects only**	*Coef*	*p*	*Coef*	*p*	*Coef*	*p*	*Coef*	*p*
Concurrent	0.844	0.060	-0.065	0.225	-0.180	0.061	1.871	0.023
5-year lag	1.866	0.003	-0.005	0.942	0.069	0.572	3.116	0.052
10-year lag	-0.415	0.761	0.302	0.070	-0.100	0.667	-1.254	0.589
Chow-test (*p*-value): net industry effects		0.010		0.172		0.214		0.035
Within R^2	0.1419		0.0257		0.0628		0.0498	
**Heterogeneous industry effects, by capacity**	*Coef*	*p*	*Coef*	*p*	*Coef*	*p*	*Coef*	*p*
Concurrent	0.734	0.279	0.095	0.325	-0.163	0.251	1.146	0.235
5-year lag	1.707	0.042	-0.069	0.466	0.170	0.344	0.877	0.578
10-year lag	0.321	0.821	0.315	0.069	-0.199	0.420	-0.486	0.838
Concurrent: Large-capacity (≥100K tons/year)	0.068	0.930	-0.236	0.018	-0.010	0.950	0.869	0.475
5-year lag: Large-capacity (≥100K tons/year)	0.451	0.703	0.149	0.219	-0.201	0.364	4.018	0.204
10-year lag: Large-capacity (≥100K tons/year)	-8.100	0.054	0.060	0.922	0.957	0.116	-5.545	0.311
Chow-test (*p*-value): main effects		0.199		0.191		0.338		0.587
Chow-test (*p*-value): heterogeneous effects		0.285		0.071		0.386		0.391
Chow-test (*p*-value): net industry effects		0.024		0.025		0.308		0.136
Within R^2	0.1420		0.0256		0.0629		0.050	

†Estimates after one-to-one propensity score matching (Caliper=0.005) without replacement and fixed-effects panel. Number of NFI plots: 14,342; number of observations=39,882.

Table S5. Wood pellet industry heterogeneous effects (manufacturing capacity) on selected C stocks within timberlands of the US coastal southeast, extended procurement radii†.

	Live trees		Standing-dead trees		Soils		Total	
**Main effects**	*Coef*	*p*	*Coef*	*p*	*Coef*	*p*	*Coef*	*p*
Concurrent	-0.176	0.673	-0.105	0.044	0.018	0.842	-0.237	0.738
5-year lag	0.875	0.104	-0.092	0.148	0.190	0.117	1.532	0.140
10-year lag	-1.098	0.299	0.142	0.299	-0.176	0.410	-2.511	0.113
Chow-test (*p*-value): main effects	0.242		0.049		0.387		0.196	
Within R^2	0.1482		0.0301		0.0549		0.0643	
**Heterogeneous industry effects, by capacity**	*Coef*	*p*	*Coef*	*p*	*Coef*	*p*	*Coef*	*p*
Concurrent	-0.274	0.672	-0.048	0.586	0.220	0.087	-0.232	0.850
5-year lag	0.165	0.820	-0.148	0.126	0.302	0.063	0.318	0.771
10-year lag	-0.684	0.533	0.162	0.248	-0.231	0.298	-2.076	0.205
Concurrent: Large-capacity (≥100K tons/year)	0.015	0.984	-0.095	0.290	-0.289	0.053	-0.184	0.882
5-year lag: Large-capacity (≥100K tons/year)	1.504	0.142	0.134	0.269	-0.171	0.426	2.505	0.142
10-year lag: Large-capacity (≥100K tons/year)	-5.862	0.102	-0.155	0.767	1.098	0.049	-5.449	0.175
Chow-test (*p*-value): main effects		0.886		0.338		0.082		0.523
Chow-test (*p*-value): heterogeneous effects		0.218		0.386		0.038		0.282
Chow-test (*p*-value): net industry effects		0.260		0.308		0.073		0.334
Within R^2	0.1483		0.0302		0.0551		0.0643	

†Estimates after one-to-one propensity score matching (Caliper=0.005) without replacement and fixed-effects panel. Number of NFI plots: 19,438; number of observations=52,895.

Table S6. Wood pellet industry heterogeneous effects (radii overlap) on selected C stocks within timberlands of the US coastal southeast, commercial procurement radii†.

	Live trees		Standing-dead trees		Soils		Total	
**Heterogeneous industry effects, by number of overlaps of industry procurement radii**	*Coef*	*p*	*Coef*	*p*	*Coef*	*p*	*Coef*	*p*
Concurrent: Within a single procurement radius	1.762	0.031	0.049	0.604	0.060	0.686	2.894	0.006
Concurrent: Within two procurement radii	1.706	0.324	-0.350	0.030	0.422	0.077	8.453	0.277
Concurrent: Within three procurement radii	3.956	0.061	-0.419	0.021	-0.692	0.057	3.745	0.138
Concurrent: Within four procurement radii	-1.345	0.781	-0.078	0.859	-1.729	0.062	-2.743	0.613
Concurrent: Within five procurement radii	20.693	0.017	-0.050	0.919	-1.871	0.222	22.769	0.015
5-year lag: Within a single procurement radius	1.061	0.115	0.039	0.592	0.027	0.837	1.963	0.182
5-year lag: Within two procurement radii	0.447	0.776	0.185	0.340	0.540	0.100	-0.493	0.838
5-year lag: Within three procurement radii	8.160	0.047	-0.272	0.467	1.125	0.022	8.235	0.073
5-year lag: Within four procurement radii	5.046	0.415	0.630	0.468	1.691	0.391	4.540	0.556
5-year lag: Within five procurement radii	-7.225	0.056	1.961	0.179	2.590	<0.001	-6.105	0.244
10-year lag: Within a single procurement radius	-0.619	0.653	0.292	0.086	-0.145	0.536	-1.850	0.437
10-year lag: Within two procurement radii	-2.961	0.708	-0.702	0.387	-0.672	0.679	-11.771	0.235
Chow-test (*p*-value): single procurement radius	0.026		0.279		0.896		0.006	
Chow-test (*p*-value): radii overlap effects	<0.001		0.123		<0.001		<0.001	
Chow-test (*p*-value): net industry effects	<0.001		0.103		<0.001		<0.001	
Within R^2	0.1426		0.0262		0.0633		0.0503	

†Estimates after one-to-one propensity score matching (Caliper=0.005) without replacement and fixed-effects panel. Number of NFI plots: 14,342; number of observations=39,882.

Table S7. Wood pellet industry heterogeneous effects (radii overlap) on selected C stocks within timberlands of the US coastal southeast, extended procurement radii†.

	Live trees		Standing dead-trees		Soils		Total	
**Heterogeneous industry effects, by number of overlaps of industry procurement radii**	*Coef*	*p*	*Coef*	*p*	*Coef*	*p*	*Coef*	*p*
Concurrent: Within a single procurement radius	0.577	0.452	-0.003	0.966	0.010	0.949	2.124	0.212
Concurrent: Within two procurement radii	-1.589	0.331	-0.188	0.267	-0.107	0.693	-1.573	0.413
Concurrent: Within three procurement radii	1.360	0.432	-0.462	0.012	0.024	0.937	0.809	0.686
Concurrent: Within four procurement radii	0.037	0.989	-0.323	0.220	-0.521	0.353	-2.488	0.383
Concurrent: Within five procurement radii	-1.531	0.738	-0.166	0.511	-0.703	0.540	-2.535	0.610
Concurrent: Within six procurement radii	-0.842	0.931	-0.306	0.122	-2.709	<0.001	-4.049	0.723
5-year lag: Within a single procurement radius	0.604	0.304	-0.071	0.307	0.164	0.212	1.276	0.228
5-year lag: Within two procurement radii	0.732	0.586	0.173	0.277	0.321	0.297	1.056	0.525
5-year lag: Within three procurement radii	3.428	0.363	0.205	0.566	0.662	0.144	4.330	0.302
5-year lag: Within four procurement radii	5.069	0.270	-0.276	0.723	2.673	0.059	6.543	0.203
5-year lag: Within five procurement radii	0.056	0.996	-1.199	0.039	1.807	0.040	0.100	0.994
5-year lag: Within six procurement radii	22.080	0.002	0.663	0.577	-0.138	0.912	25.636	0.002
10-year lag: Within a single procurement radius	-0.985	0.357	0.134	0.350	-0.224	0.308	-2.517	0.122
10-year lag: Within two procurement radii	-7.793	0.161	-0.843	0.058	-0.562	0.536	-14.543	0.029
Chow-test (*p*-value): single procurement radius	0.385		0.505		0.461		0.226	
Chow-test (*p*-value): radii overlap effects	0.620		0.020		0.166		0.358	
Chow-test (*p*-value): net industry effects	0.667		0.007		0.314		0.406	
Within R^2	0.1487		0.0304		0.0552		0.0646	

†Estimates after one-to-one propensity score matching (Caliper=0.005) without replacement and fixed-effects panel. Number of NFI plots: 19,438; number of observations=52,895

A)

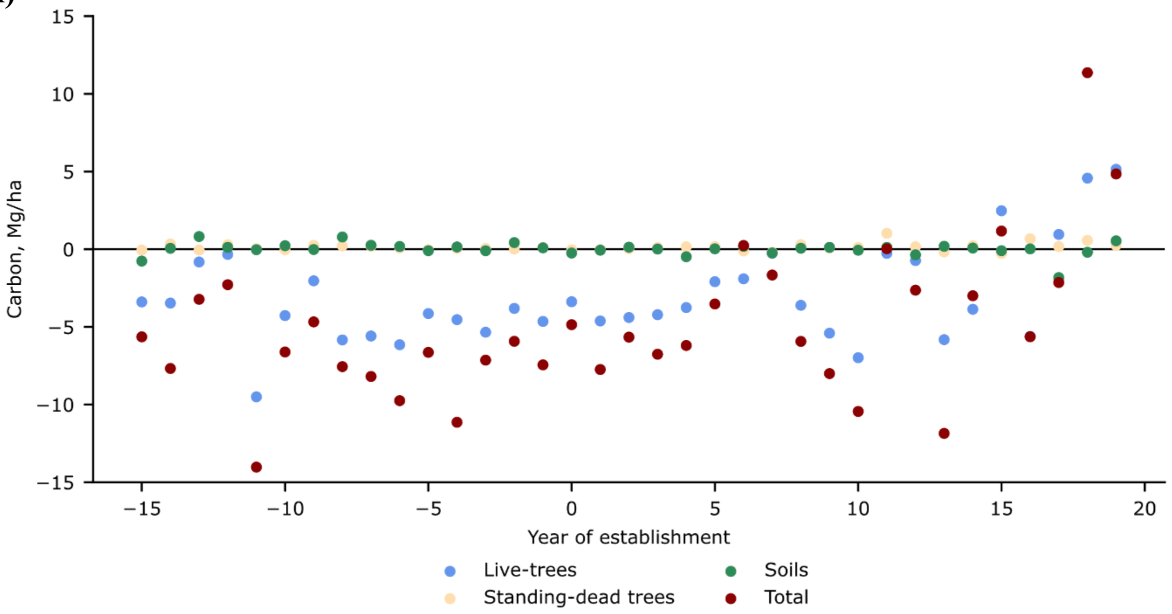

B)

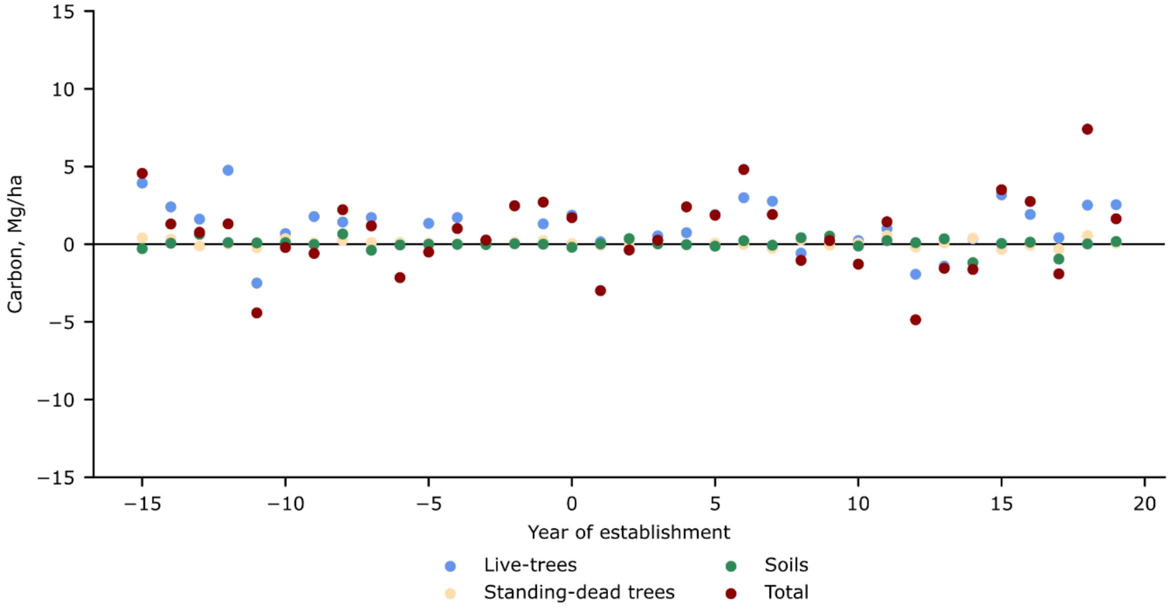

Fig. S1. Estimated coefficients from event study design to test parallel trends in treatment and control areas prior to treatment by (**A**) commercial and (**B**) extended procurement radii.

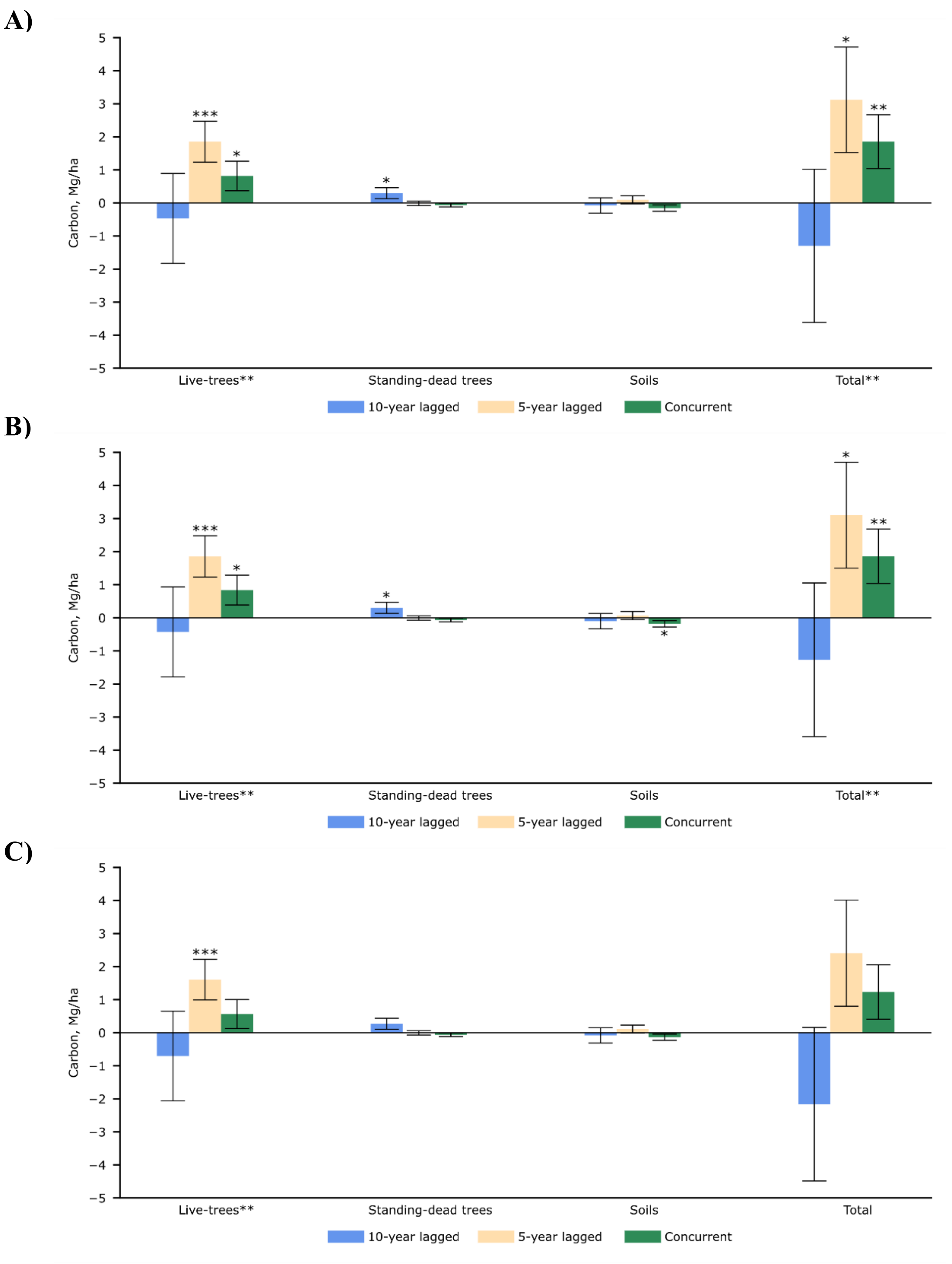

Fig. S2. Estimated average concurrent and lagged effects of the wood pellet industry on selected C stocks in timberlands of the US coastal southeast using different matching algorithms (commercial procurement radii).: (A) Nearest-neighbor, (B) One-to-one, caliper=0.01, (C) One-to-two, caliper= 0.00005. Bars denote forest inventory plot cluster-level robust standard errors. Type-I errors (*<0.10, **<0.05, ***<0.01) of concurrent, lagged, and net effects on respective carbon stocks.

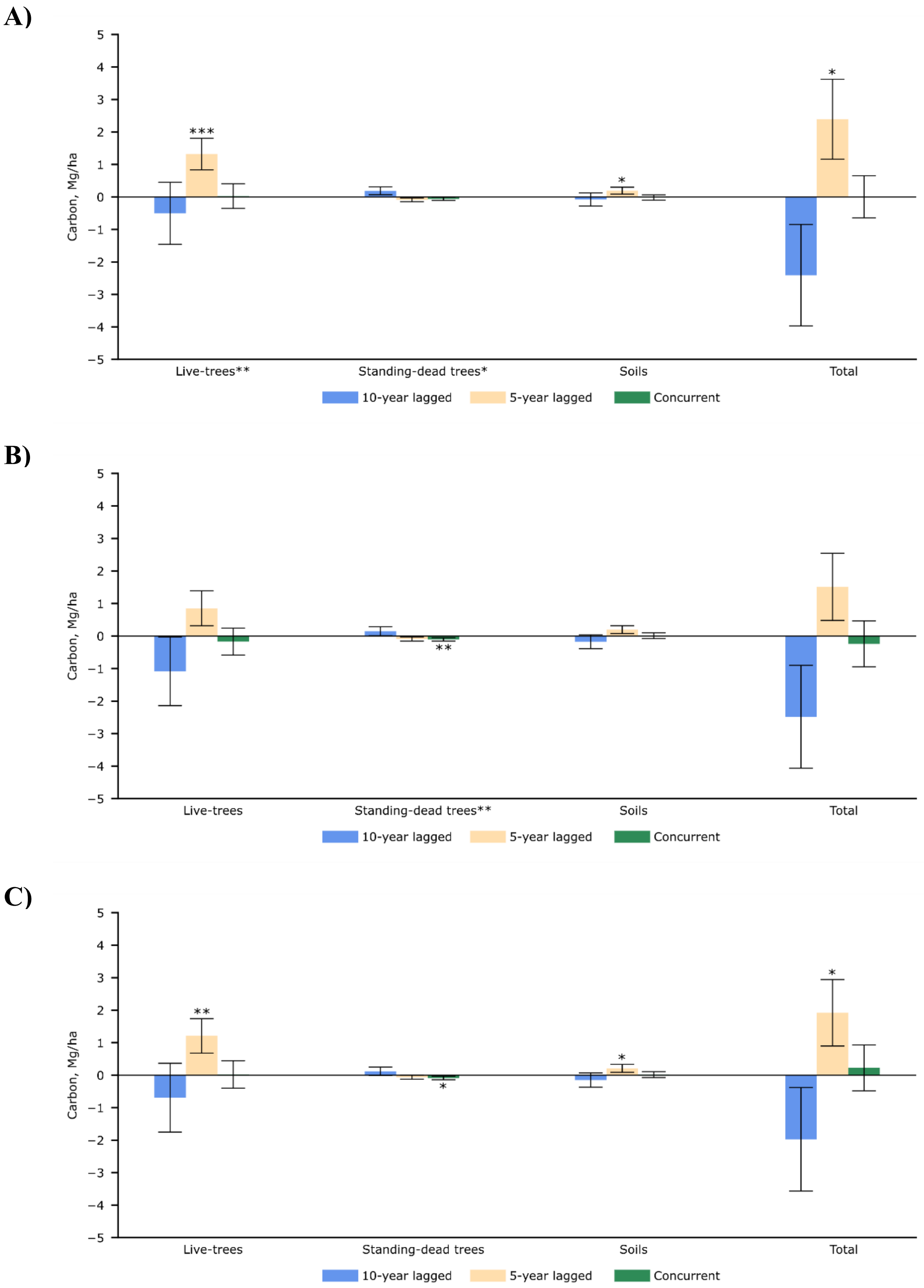

Fig. S3. Estimated average concurrent and lagged effects of the wood pellet industry on selected C stocks in timberlands of the US coastal southeast using different matching algorithms (extended procurement radii): (**A**) Nearest-neighbor, (**B**) One-to-one, caliper=0.01, (**C**) One-to-two, caliper= 0.00005. Bars denote NFI plot cluster-level robust standard errors. Type-I errors (*<0.10, **<0.05, ***<0.01) of concurrent, lagged, and net effects on respective carbon stocks.

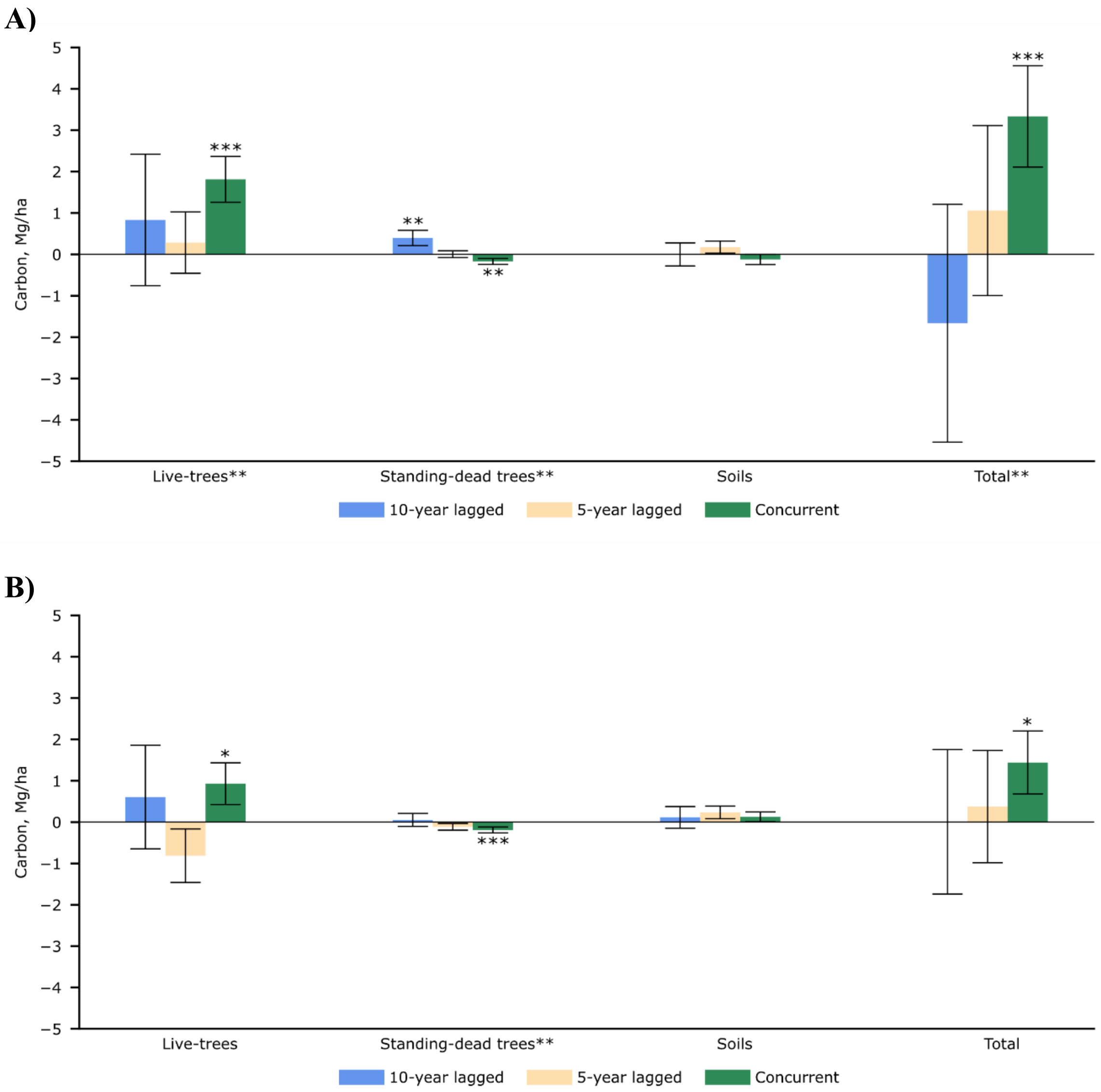

Fig. S4. Estimated average concurrent and lagged effects of the wood pellet industry on selected C stocks within timberlands of the states of Alabama, Georgia, and Virginia. Estimates by (**A**) commercial and (**B**) extended procurement radii. Coefficients inferred after one-to-one propensity score matching (Caliper=0.005) without replacement and fixed-effects panel. Bars denote NFI plot cluster-level robust standard errors. Type-I errors (*<0.10, **<0.05, ***<0.01) of concurrent, lagged, and net effects on respective carbon stocks.

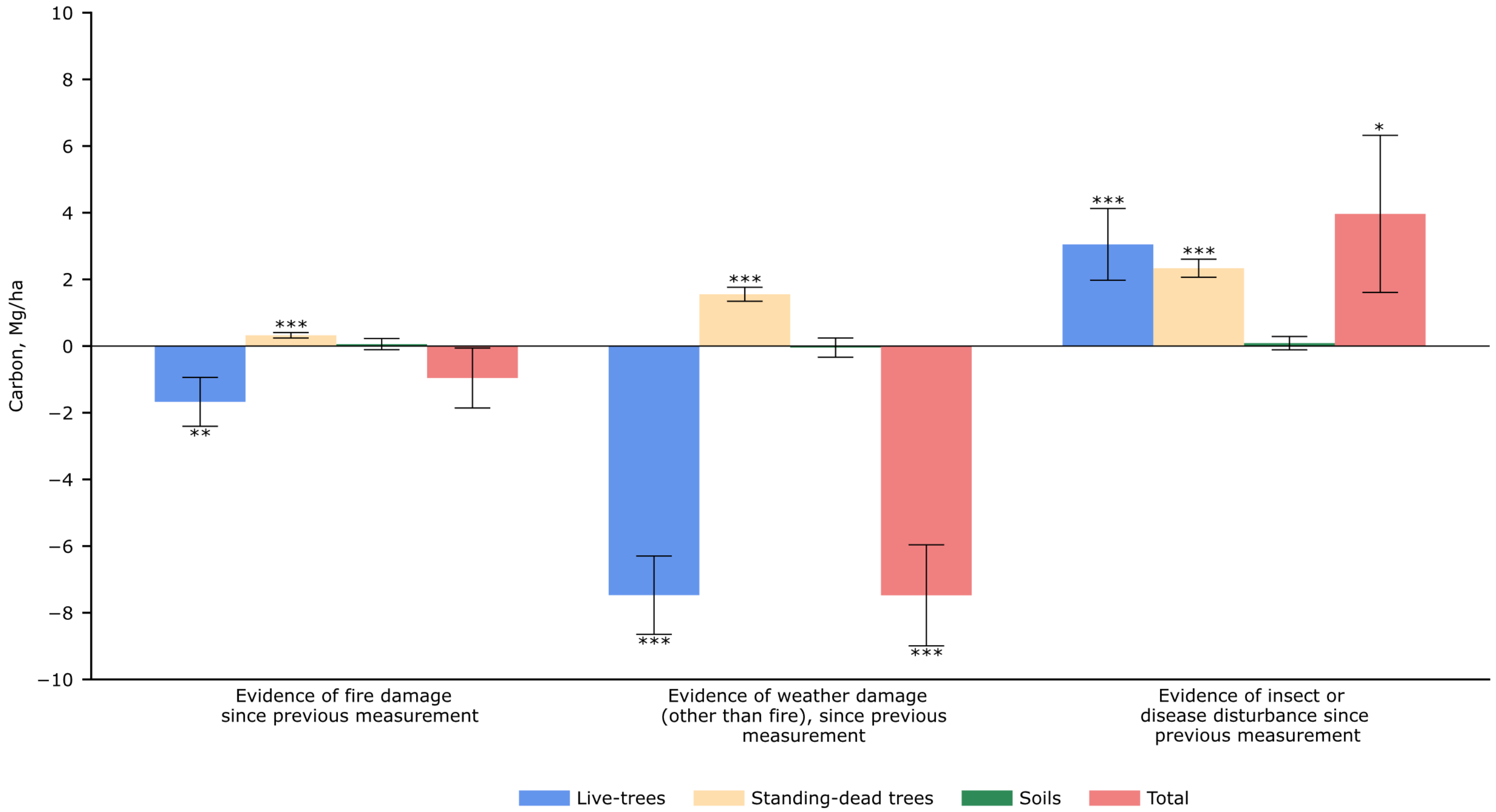

Fig. S5. Estimated associations between selected abiotic and biotic factors and C stocks in timberlands of the US coastal southeast within commercial procurement radii [NFI plots=14,342; Observations= 39,882], inferred after propensity score matching (caliper= 0.005, without replacement) and fixed-effects panel regression. Bars denote NFI plot cluster-level robust standard errors. Type-I errors: *<0.10, **<0.05, ***<0.01).

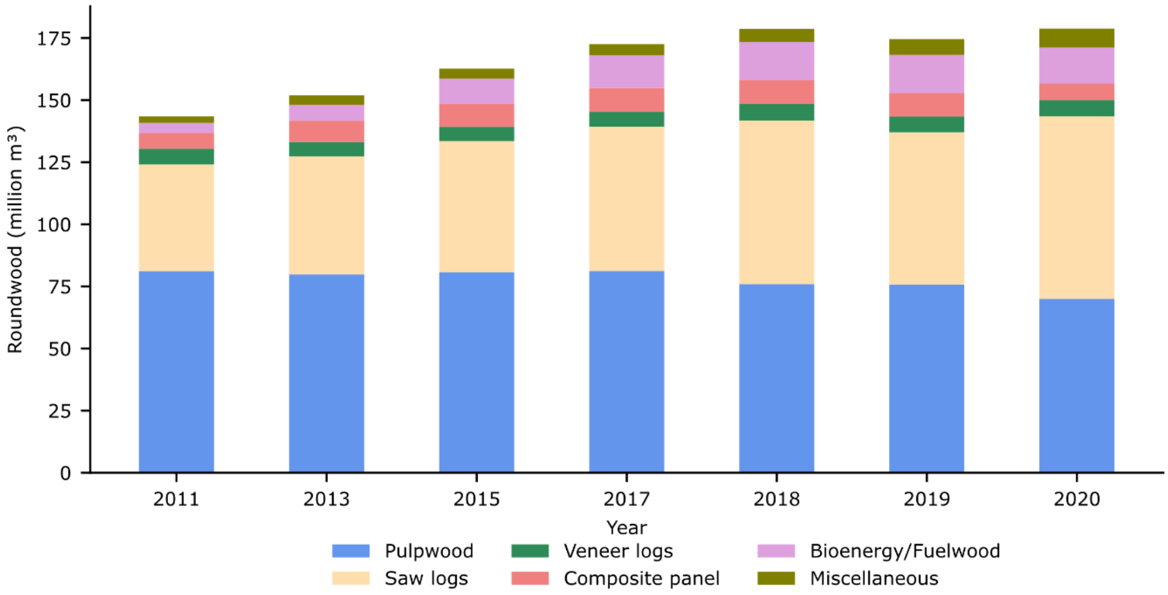

Fig. S6. Roundwood production utilization, by product type in the US coastal southeast, 2011-2020. Source: Adapted from **[33]**.
